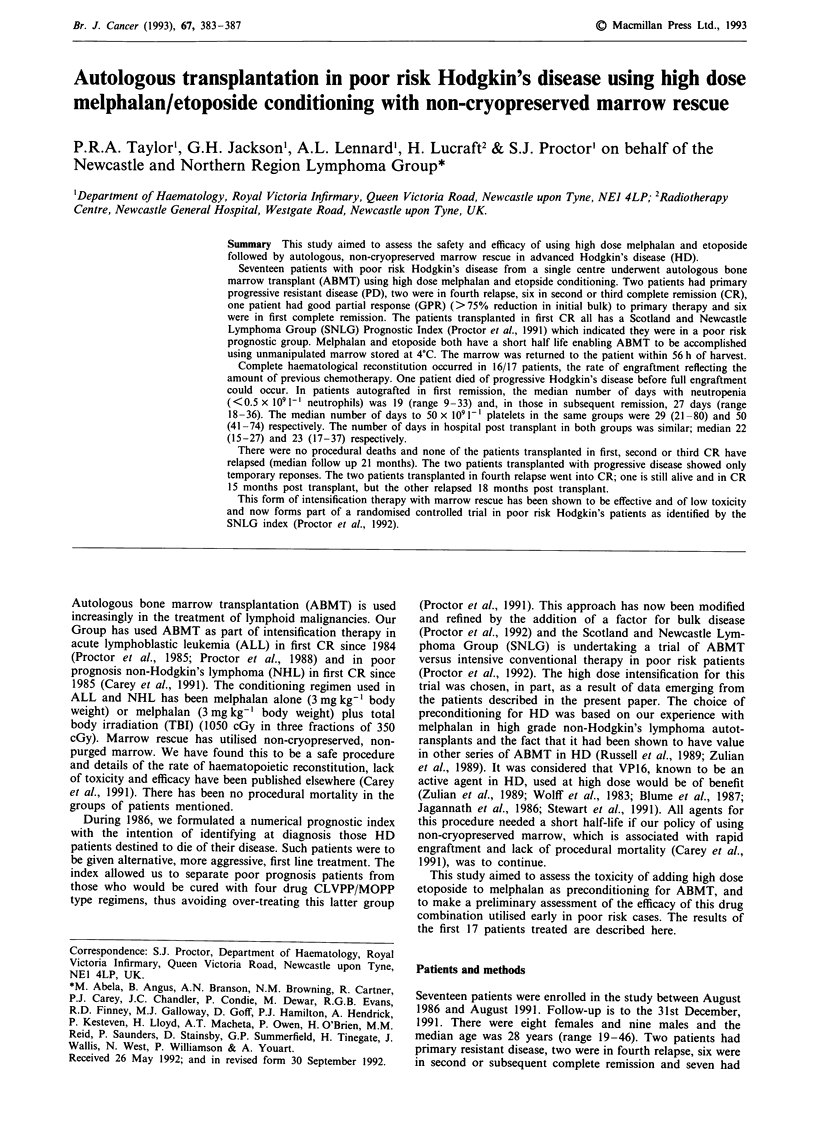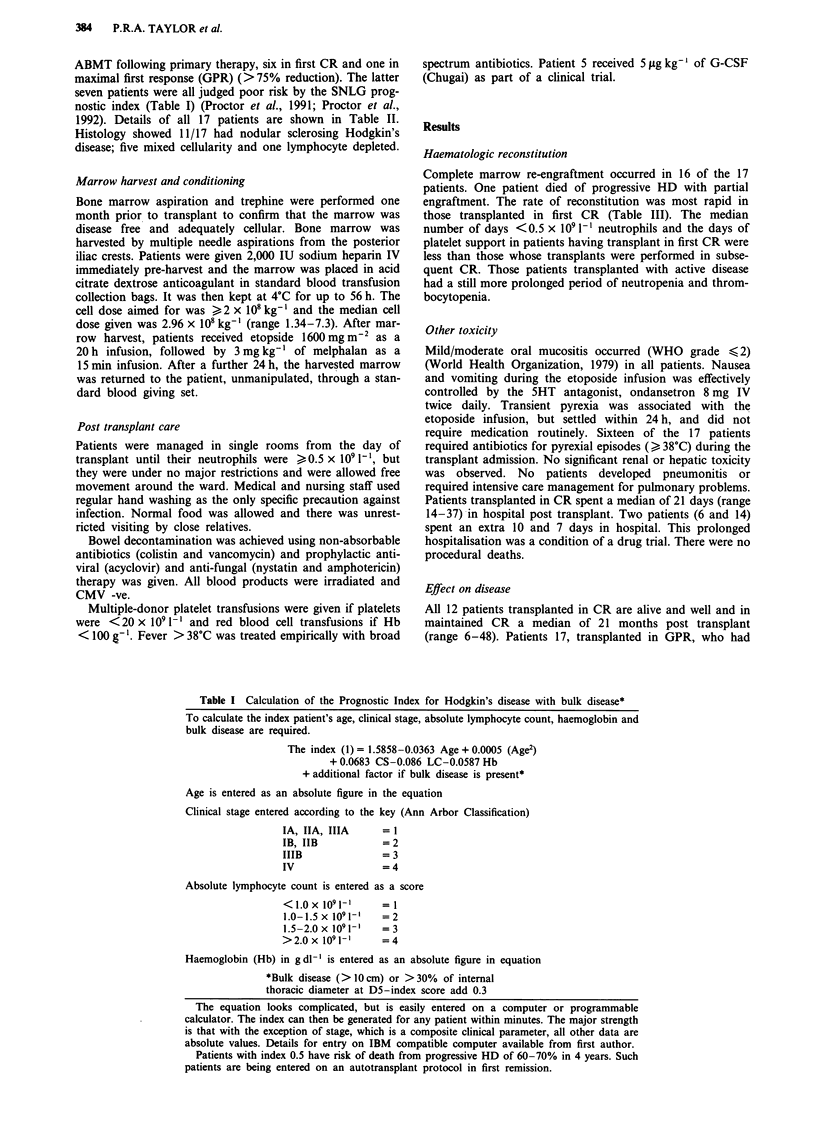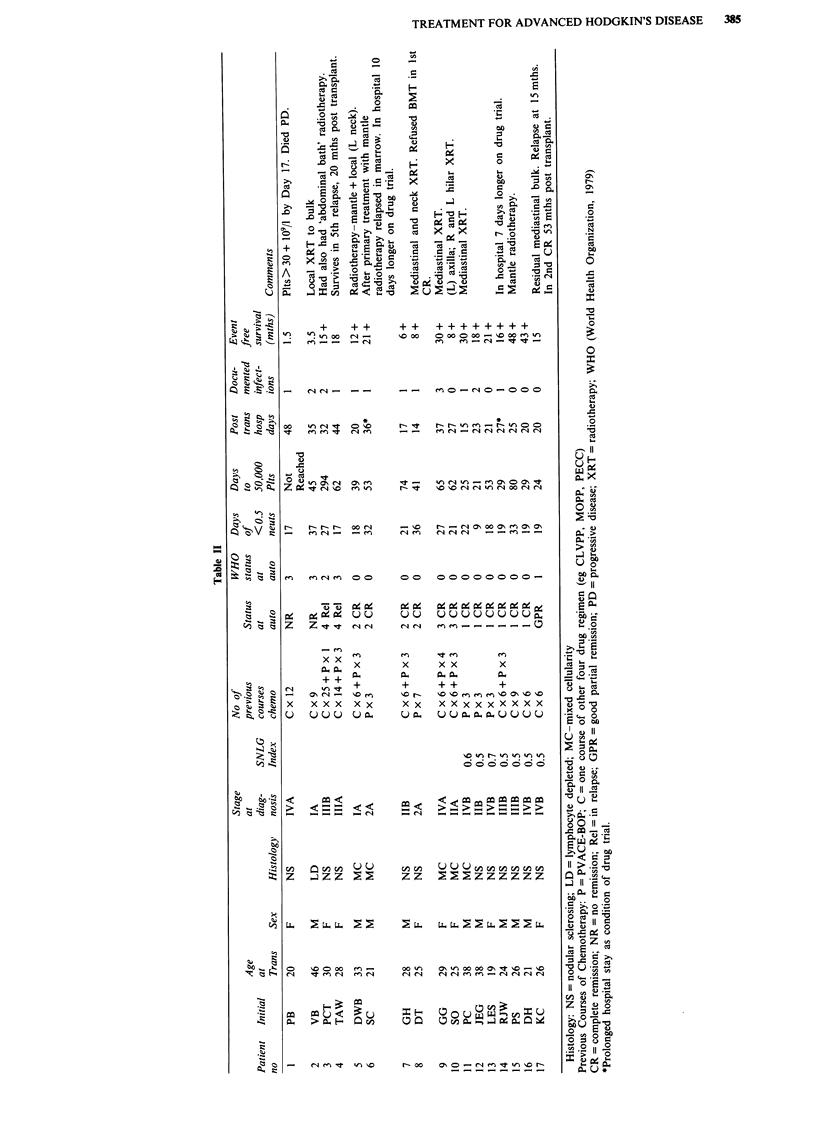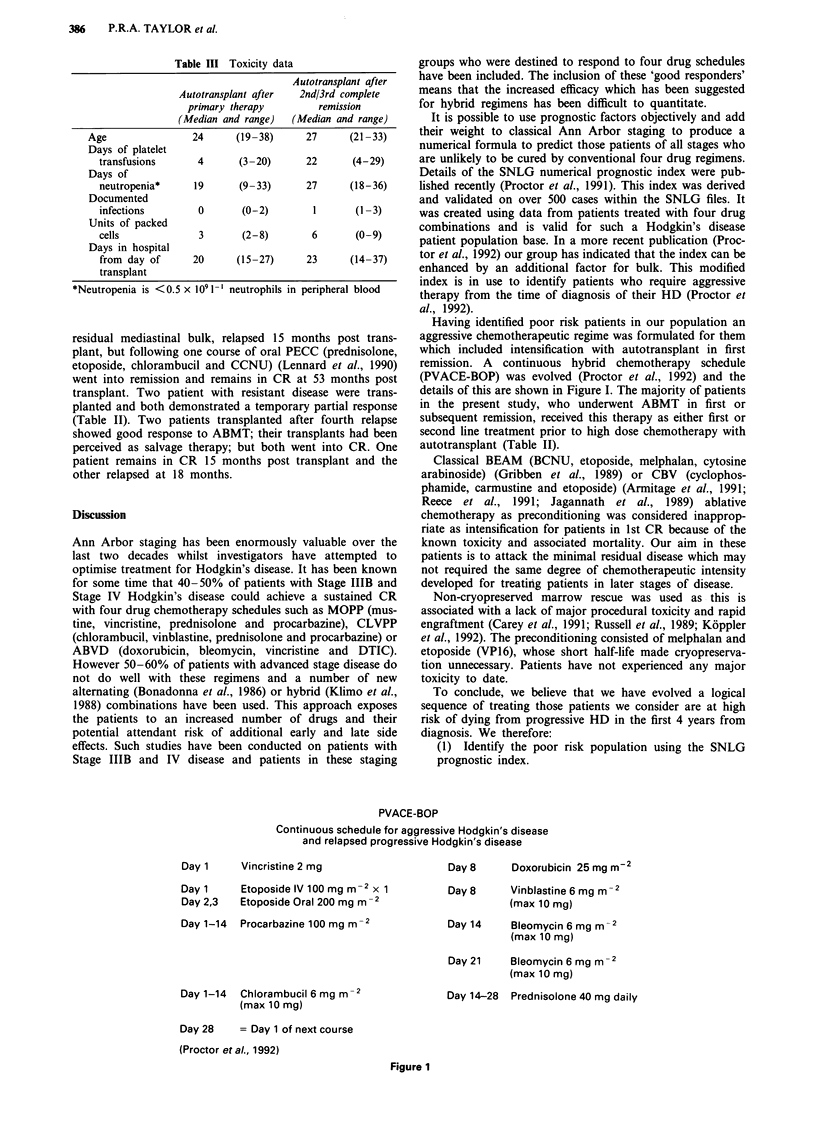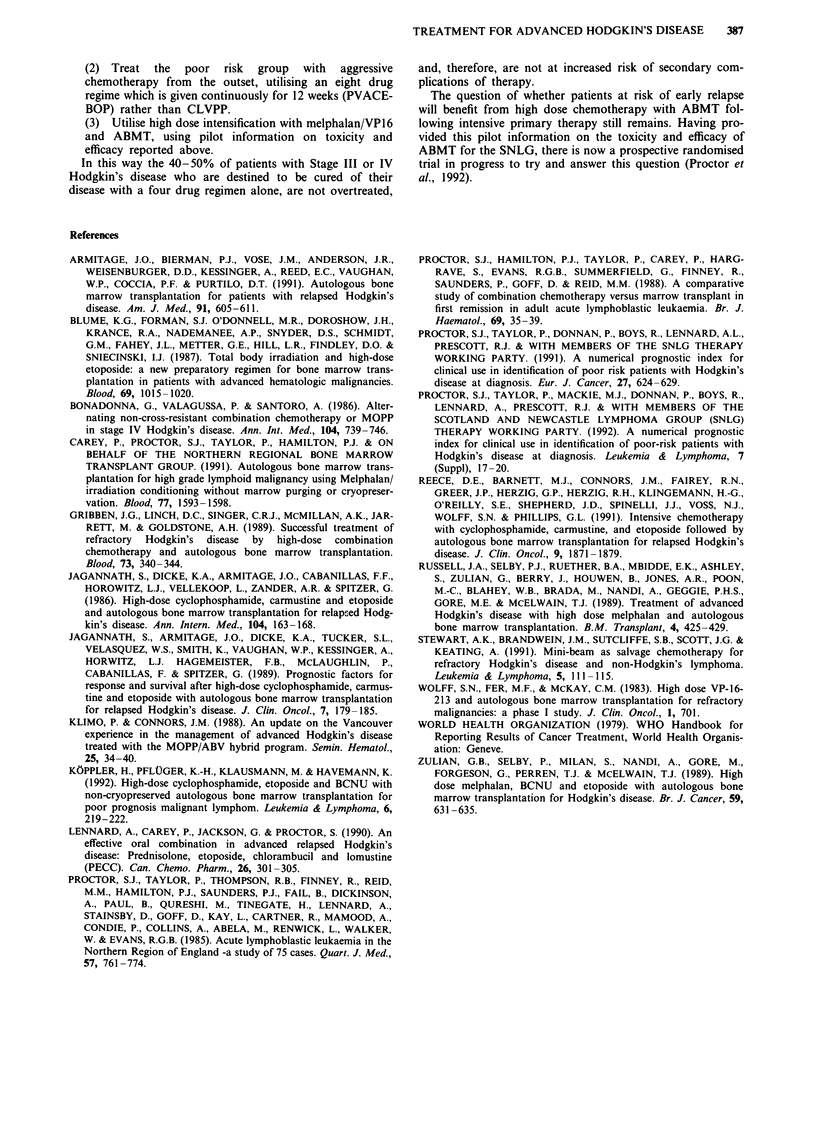# Autologous transplantation in poor risk Hodgkin's disease using high dose melphalan/etoposide conditioning with non-cryopreserved marrow rescue. The Newcastle and Northern Region Lymphoma Group.

**DOI:** 10.1038/bjc.1993.70

**Published:** 1993-02

**Authors:** P. R. Taylor, G. H. Jackson, A. L. Lennard, H. Lucraft, S. J. Proctor

**Affiliations:** Department of Haematology, Royal Victoria Infirmary, Newcastle upon Tyne, UK.

## Abstract

This study aimed to assess the safety and efficacy of using high dose melphalan and etoposide followed by autologous, non-cryopreserved marrow rescue in advanced Hodgkin's disease (HD). Seventeen patients with poor risk Hodgkin's disease from a single centre underwent autologous bone marrow transplant (ABMT) using high dose melphalan and etopside conditioning. Two patients had primary progressive resistant disease (PD), two were in fourth relapse, six in second or third complete remission (CR), one patient had good partial response (GPR) (> 75% reduction in initial bulk) to primary therapy and six were in first complete remission. The patients transplanted in first CR all has a Scotland and Newcastle Lymphoma Group (SNLG) Prognostic Index (Proctor et al., 1991) which indicated they were in a poor risk prognostic group. Melphalan and etoposide both have a short half life enabling ABMT to be accomplished using unmanipulated marrow stored at 4 degrees C. The marrow was returned to the patient within 56 h of harvest. Complete haematological reconstitution occurred in 16/17 patients, the rate of engraftment reflecting the amount of previous chemotherapy. One patient died of progressive Hodgkin's disease before full engraftment could occur. In patients autografted in first remission, the median number of days with neutropenia (< 0.5 x 10(9) l-1 neutrophils) was 19 (range 9-33) and, in those in subsequent remission, 27 days (range 18-36). The median number of days to 50 x 10(9) l-1 platelets in the same groups were 29 (21-80) and 50 (41-74) respectively. The number of days in hospital post transplant in both groups was similar; median 22 (15-27) and 23 (17-37) respectively. There were no procedural deaths and none of the patients transplanted in first, second or third CR have relapsed (median follow up 21 months). The two patients transplanted with progressive disease showed only temporary responses. The two patients transplanted in fourth relapse went into CR; one is still alive and in CR 15 months post transplant, but the other relapsed 18 months post transplant. This form of intensification therapy with marrow rescue has been shown to be effective and of low toxicity and now forms part of a randomised controlled trial in poor risk Hodgkin's patients as identified by the SNLG index (Proctor et al., 1992).


 © Macmillan Press Ltd., 1993

### Autologous transplantation in poor risk Hodgkin's disease using high dose melphalan/etoposide conditioning with non-cryopreserved marrow rescue

P.R.A. Taylor[1], G.H. Jackson[1], A.L. Lennard[1], H. Lucraft[2] & S.J. Proctor[1] on behalf of the Newcastle and Northern Region Lymphoma Group*

[1]*Department of Haematology, Royal Victoria Infirmary, Queen Victoria Road, Newcastle upon Tyne, NE1 4LP;* [2]*Radiotherapy Centre, Newcastle General Hospital, Westgate Road, Newcastle upon Tyne, UK.*

**Summary** This study aimed to assess the safety and efficacy of using high dose melphalan and etoposide followed by autologous, non-cryopreserved marrow rescue in advanced Hodgkin's disease (HD).

Seventeen patients with poor risk Hodgkin's disease from a single centre underwent autologous bone marrow transplant (ABMT) using high dose melphalan and etopside conditioning. Two patients had primary progressive resistant disease (PD), two were in fourth relapse, six in second or third complete remission (CR), one patient had good partial response (GPR) (>75% reduction in initial bulk) to primary therapy and six were in first complete remission. The patients transplanted in first CR all has a Scotland and Newcastle Lymphoma Group (SNLG) Prognostic Index (Proctor *et al.*, 1991) which indicated they were in a poor risk prognostic group. Melphalan and etoposide both have a short half life enabling ABMT to be accomplished using unmanipulated marrow stored at 4°C. The marrow was returned to the patient within 56 h of harvest.

Complete haematological reconstitution occurred in 16/17 patients, the rate of engraftment reflecting the amount of previous chemotherapy. One patient died of progressive Hodgkin's disease before full engraftment could occur. In patients autografted in first remission, the median number of days with neutropenia ($<0.5 \times 10^9 l^{-1}$ neutrophils) was 19 (range 9–33) and, in those in subsequent remission, 27 days (range 18–36). The median number of days to $50 \times 10^9 l^{-1}$ platelets in the same groups were 29 (21–80) and 50 (41–74) respectively. The number of days in hospital post transplant in both groups was similar; median 22 (15–27) and 23 (17–37) respectively.

There were no procedural deaths and none of the patients transplanted in first, second or third CR have relapsed (median follow up 21 months). The two patients transplanted with progressive disease showed only temporary reponses. The two patients transplanted in fourth relapse went into CR; one is still alive and in CR 15 months post transplant, but the other relapsed 18 months post transplant.

This form of intensification therapy with marrow rescue has been shown to be effective and of low toxicity and now forms part of a randomised controlled trial in poor risk Hodgkin's patients as identified by the SNLG index (Proctor *et al.*, 1992).

Autologous bone marrow transplantation (ABMT) is used increasingly in the treatment of lymphoid malignancies. Our Group has used ABMT as part of intensification therapy in acute lymphoblastic leukemia (ALL) in first CR since 1984 (Proctor *et al.*, 1985; Proctor *et al.*, 1988) and in poor prognosis non-Hodgkin's lymphoma (NHL) in first CR since 1985 (Carey *et al.*, 1991). The conditioning regimen used in ALL and NHL has been melphalan alone ($3 mg kg^{-1}$ body weight) or melphalan ($3 mg kg^{-1}$ body weight) plus total body irradiation (TBI) (1050 cGy in three fractions of 350 cGy). Marrow rescue has utilised non-cryopreserved, non-purged marrow. We have found this to be a safe procedure and details of the rate of haematopoietic reconstitution, lack of toxicity and efficacy have been published elsewhere (Carey *et al.*, 1991). There has been no procedural mortality in the groups of patients mentioned.

During 1986, we formulated a numerical prognostic index with the intention of identifying at diagnosis those HD patients destined to die of their disease. Such patients were to be given alternative, more aggressive, first line treatment. The index allowed us to separate poor prognosis patients from those who would be cured with four drug CLVPP/MOPP type regimens, thus avoiding over-treating this latter group (Proctor *et al.*, 1991). This approach has now been modified and refined by the addition of a factor for bulk disease (Proctor *et al.*, 1992) and the Scotland and Newcastle Lymphoma Group (SNLG) is undertaking a trial of ABMT versus intensive conventional therapy in poor risk patients (Proctor *et al.*, 1992). The high dose intensification for this trial was chosen, in part, as a result of data emerging from the patients described in the present paper. The choice of preconditioning for HD was based on our experience with melphalan in high grade non-Hodgkin's lymphoma autotransplants and the fact that it had been shown to have value in other series of ABMT in HD (Russell *et al.*, 1989; Zulian *et al.*, 1989). It was considered that VP16, known to be an active agent in HD, used at high dose would be of benefit (Zulian *et al.*, 1989; Wolff *et al.*, 1983; Blume *et al.*, 1987; Jagannath *et al.*, 1986; Stewart *et al.*, 1991). All agents for this procedure needed a short half-life if our policy of using non-cryopreserved marrow, which is associated with rapid engraftment and lack of procedural mortality (Carey *et al.*, 1991), was to continue.

This study aimed to assess the toxicity of adding high dose etoposide to melphalan as preconditioning for ABMT, and to make a preliminary assessment of the efficacy of this drug combination utilised early in poor risk cases. The results of the first 17 patients treated are described here.

### Patients and methods

Seventeen patients were enrolled in the study between August 1986 and August 1991. Follow-up is to the 31st December, 1991. There were eight females and nine males and the median age was 28 years (range 19–46). Two patients had primary resistant disease, two were in fourth relapse, six were in second or subsequent complete remission and seven had

Correspondence: S.J. Proctor, Department of Haematology, Royal Victoria Infirmary, Queen Victoria Road, Newcastle upon Tyne, NE1 4LP, UK.

*M. Abela, B. Angus, A.N. Branson, N.M. Browning, R. Cartner, P.J. Carey, J.C. Chandler, P. Condie, M. Dewar, R.G.B. Evans, R.D. Finney, M.J. Galloway, D. Goff, P.J. Hamilton, A. Hendrick, P. Kesteven, H. Lloyd, A.T. Macheta, P. Owen, H. O'Brien, M.M. Reid, P. Saunders, D. Stainsby, G.P. Summerfield, H. Tinegate, J. Wallis, N. West, P. Williamson & A. Youart.

Received 26 May 1992; and in revised form 30 September 1992.

ABMT following primary therapy, six in first CR and one in maximal first response (GPR) (>75% reduction). The latter seven patients were all judged poor risk by the SNLG prognostic index (Table I) (Proctor et al., 1991; Proctor et al., 1992). Details of all 17 patients are shown in Table II. Histology showed 11/17 had nodular sclerosing Hodgkin's disease; five mixed cellularity and one lymphocyte depleted.

*Marrow harvest and conditioning*

Bone marrow aspiration and trephine were performed one month prior to transplant to confirm that the marrow was disease free and adequately cellular. Bone marrow was harvested by multiple needle aspirations from the posterior iliac crests. Patients were given 2,000 IU sodium heparin IV immediately pre-harvest and the marrow was placed in acid citrate dextrose anticoagulant in standard blood transfusion collection bags. It was then kept at 4°C for up to 56 h. The cell dose aimed for was $\geqslant 2 \times 10^8$ kg$^{-1}$ and the median cell dose given was $2.96 \times 10^8$ kg$^{-1}$ (range 1.34–7.3). After marrow harvest, patients received etopside 1600 mg m$^{-2}$ as a 20 h infusion, followed by 3 mg kg$^{-1}$ of melphalan as a 15 min infusion. After a further 24 h, the harvested marrow was returned to the patient, unmanipulated, through a standard blood giving set.

*Post transplant care*

Patients were managed in single rooms from the day of transplant until their neutrophils were $\geqslant 0.5 \times 10^9$ l$^{-1}$, but they were under no major restrictions and were allowed free movement around the ward. Medical and nursing staff used regular hand washing as the only specific precaution against infection. Normal food was allowed and there was unrestricted visiting by close relatives.

Bowel decontamination was achieved using non-absorbable antibiotics (colistin and vancomycin) and prophylactic antiviral (acyclovir) and anti-fungal (nystatin and amphotericin) therapy was given. All blood products were irradiated and CMV -ve.

Multiple-donor platelet transfusions were given if platelets were $<20 \times 10^9$ l$^{-1}$ and red blood cell transfusions if Hb $<100$ g$^{-1}$. Fever >38°C was treated empirically with broad spectrum antibiotics. Patient 5 received 5 μg kg$^{-1}$ of G-CSF (Chugai) as part of a clinical trial.

### Results

*Haematologic reconstitution*

Complete marrow re-engraftment occurred in 16 of the 17 patients. One patient died of progressive HD with partial engraftment. The rate of reconstitution was most rapid in those transplanted in first CR (Table III). The median number of days $<0.5 \times 10^9$ l$^{-1}$ neutrophils and the days of platelet support in patients having transplant in first CR were less than those whose transplants were performed in subsequent CR. Those patients transplanted with active disease had a still more prolonged period of neutropenia and thrombocytopenia.

*Other toxicity*

Mild/moderate oral mucositis occurred (WHO grade $\leqslant 2$) (World Health Organization, 1979) in all patients. Nausea and vomiting during the etoposide infusion was effectively controlled by the 5HT antagonist, ondansetron 8 mg IV twice daily. Transient pyrexia was associated with the etoposide infusion, but settled within 24 h, and did not require medication routinely. Sixteen of the 17 patients required antibiotics for pyrexial episodes ($\geqslant$38°C) during the transplant admission. No significant renal or hepatic toxicity was observed. No patients developed pneumonitis or required intensive care management for pulmonary problems. Patients transplanted in CR spent a median of 21 days (range 14–37) in hospital post transplant. Two patients (6 and 14) spent an extra 10 and 7 days in hospital. This prolonged hospitalisation was a condition of a drug trial. There were no procedural deaths.

*Effect on disease*

All 12 patients transplanted in CR are alive and well and in maintained CR a median of 21 months post transplant (range 6–48). Patients 17, transplanted in GPR, who had

**Table I** Calculation of the Prognostic Index for Hodgkin's disease with bulk disease*

To calculate the index patient's age, clinical stage, absolute lymphocyte count, haemoglobin and bulk disease are required.

$$\text{The index (1)} = 1.5858 - 0.0363\ \text{Age} + 0.0005\ (\text{Age}^2) + 0.0683\ \text{CS} - 0.086\ \text{LC} - 0.0587\ \text{Hb}$$
+ additional factor if bulk disease is present*

Age is entered as an absolute figure in the equation

Clinical stage entered according to the key (Ann Arbor Classification)

| | |
|---|---|
| IA, IIA, IIIA | = 1 |
| IB, IIB | = 2 |
| IIIB | = 3 |
| IV | = 4 |

Absolute lymphocyte count is entered as a score

| | |
|---|---|
| $<1.0 \times 10^9$ l$^{-1}$ | = 1 |
| $1.0-1.5 \times 10^9$ l$^{-1}$ | = 2 |
| $1.5-2.0 \times 10^9$ l$^{-1}$ | = 3 |
| $>2.0 \times 10^9$ l$^{-1}$ | = 4 |

Haemoglobin (Hb) in g dl$^{-1}$ is entered as an absolute figure in equation

*Bulk disease (>10 cm) or >30% of internal thoracic diameter at D5-index score add 0.3

The equation looks complicated, but is easily entered on a computer or programmable calculator. The index can then be generated for any patient within minutes. The major strength is that with the exception of stage, which is a composite clinical parameter, all other data are absolute values. Details for entry on IBM compatible computer available from first author.

Patients with index 0.5 have risk of death from progressive HD of 60–70% in 4 years. Such patients are being entered on an autotransplant protocol in first remission.

**Table II**

| *Patient no* | *Initial* | *Age at Trans* | *Sex* | *Histology* | *Stage at diag-nosis* | *SNLG Index* | *No of previous courses chemo* | *Status at auto* | *WHO status at auto* | *Days of <0.5 neuts* | *Days to 50,000 Plts* | *Post trans hosp days* | *Docu-mented infect-ions* | *Event free survival (mths)* | *Comments* |
|---|---|---|---|---|---|---|---|---|---|---|---|---|---|---|---|
| 1 | PB | 20 | F | NS | IVA | | C × 12 | NR | 3 | 17 | Not Reached | 48 | 1 | 1.5 | Plts > 30 + $10^9$/l by Day 17. Died PD. |
| 2 | VB | 46 | M | LD | IA | | C × 9 | NR | 3 | 37 | 45 | 35 | 2 | 3.5 | Local XRT to bulk |
| 3 | PCT | 30 | F | NS | IIIB | | C × 25 + P × 1 | 4 Rel | 2 | 27 | 294 | 32 | 2 | 15 + | Had also had 'abdominal bath' radiotherapy. |
| 4 | TAW | 28 | F | NS | IIIA | | C × 14 + P × 3 | 4 Rel | 3 | 17 | 62 | 44 | 1 | 18 | Survives in 5th relapse, 20 mths post transplant. |
| 5 | DWB | 33 | M | MC | IA | | C × 6 + P × 3 | 2 CR | 0 | 18 | 39 | 20 | 1 | 12 + | Radiotherapy-mantle + local (L neck). |
| 6 | SC | 21 | M | MC | 2A | | P × 3 | 2 CR | 0 | 32 | 53 | 36* | 1 | 21 + | After primary treatment with mantle radiotherapy relapsed in marrow. In hospital 10 days longer on drug trial. |
| 7 | GH | 28 | M | NS | IIB | | C × 6 + P × 3 | 2 CR | 0 | 21 | 74 | 17 | 1 | 6 + | |
| 8 | DT | 25 | F | NS | 2A | | P × 7 | 2 CR | 0 | 36 | 41 | 14 | 1 | 8 + | Mediastinal and neck XRT. Refused BMT in 1st CR. |
| 9 | GG | 29 | F | MC | IVA | | C × 6 + P × 4 | 3 CR | 0 | 27 | 65 | 37 | 3 | 30 + | Mediastinal XRT. |
| 10 | SO | 25 | F | MC | IIA | | C × 6 + P × 3 | 3 CR | 0 | 21 | 62 | 27 | 0 | 8 + | (L) axilla; R and L hilar XRT. |
| 11 | PC | 38 | M | MC | IVB | 0.6 | P × 3 | 1 CR | 0 | 22 | 25 | 15 | 1 | 30 + | Mediastinal XRT. |
| 12 | JEG | 38 | M | NS | IIB | 0.5 | P × 3 | 1 CR | 0 | 9 | 21 | 23 | 2 | 18 + | |
| 13 | LES | 19 | F | NS | IVB | 0.7 | P × 3 | 1 CR | 0 | 18 | 53 | 21 | 0 | 21 + | |
| 14 | RJW | 24 | M | NS | IIIB | 0.5 | C × 6 + P × 3 | 1 CR | 0 | 19 | 29 | 27* | 1 | 16 + | In hospital 7 days longer on drug trial. |
| 15 | PS | 26 | M | NS | IIIB | 0.5 | C × 9 | 1 CR | 0 | 33 | 80 | 25 | 0 | 48 + | Mantle radiotherapy. |
| 16 | DH | 21 | M | NS | IVB | 0.5 | C × 6 | 1 CR | 0 | 19 | 29 | 20 | 0 | 43 + | |
| 17 | KC | 26 | F | NS | IVB | 0.5 | C × 6 | GPR | 1 | 19 | 24 | 20 | 0 | 15 | Residual mediastinal bulk. Relapse at 15 mths. In 2nd CR 53 mths post transplant. |

Histology: NS = nodular sclerosing; LD = lymphocyte depleted; MC-mixed cellularity

Previous Courses of Chemotherapy: P = PVACE-BOP; C = one course of other four drug regimen (eg CLVPP, MOPP, PECC)

CR = complete remission; NR = no remission; Rel = in relapse; GPR = good partial remission; PD = progressive disease; XRT = radiotherapy; WHO (World Health Organization, 1979)

*Prolonged hospital stay as condition of drug trial.

**Table III** Toxicity data

| | *Autotransplant after primary therapy (Median and range)* | | *Autotransplant after 2nd/3rd complete remission (Median and range)* | |
|---|---|---|---|---|
| Age | 24 | (19–38) | 27 | (21–33) |
| Days of platelet transfusions | 4 | (3–20) | 22 | (4–29) |
| Days of neutropenia* | 19 | (9–33) | 27 | (18–36) |
| Documented infections | 0 | (0–2) | 1 | (1–3) |
| Units of packed cells | 3 | (2–8) | 6 | (0–9) |
| Days in hospital from day of transplant | 20 | (15–27) | 23 | (14–37) |

*Neutropenia is $<0.5 \times 10^9 \, l^{-1}$ neutrophils in peripheral blood residual mediastinal bulk, relapsed 15 months post transplant, but following one course of oral PECC (prednisolone, etoposide, chlorambucil and CCNU) (Lennard *et al.*, 1990) went into remission and remains in CR at 53 months post transplant. Two patient with resistant disease were transplanted and both demonstrated a temporary partial response (Table II). Two patients transplanted after fourth relapse showed good response to ABMT; their transplants had been perceived as salvage therapy; but both went into CR. One patient remains in CR 15 months post transplant and the other relapsed at 18 months.

### Discussion

Ann Arbor staging has been enormously valuable over the last two decades whilst investigators have attempted to optimise treatment for Hodgkin's disease. It has been known for some time that 40–50% of patients with Stage IIIB and Stage IV Hodgkin's disease could achieve a sustained CR with four drug chemotherapy schedules such as MOPP (mustine, vincristine, prednisolone and procarbazine), CLVPP (chlorambucil, vinblastine, prednisolone and procarbazine) or ABVD (doxorubicin, bleomycin, vincristine and DTIC). However 50–60% of patients with advanced stage disease do not do well with these regimens and a number of new alternating (Bonadonna *et al.*, 1986) or hybrid (Klimo *et al.*, 1988) combinations have been used. This approach exposes the patients to an increased number of drugs and their potential attendant risk of additional early and late side effects. Such studies have been conducted on patients with Stage IIIB and IV disease and patients in these staging groups who were destined to respond to four drug schedules have been included. The inclusion of these 'good responders' means that the increased efficacy which has been suggested for hybrid regimens has been difficult to quantitate.

It is possible to use prognostic factors objectively and add their weight to classical Ann Arbor staging to produce a numerical formula to predict those patients of all stages who are unlikely to be cured by conventional four drug regimens. Details of the SNLG numerical prognostic index were published recently (Proctor *et al.*, 1991). This index was derived and validated on over 500 cases within the SNLG files. It was created using data from patients treated with four drug combinations and is valid for such a Hodgkin's disease patient population base. In a more recent publication (Proctor *et al.*, 1992) our group has indicated that the index can be enhanced by an additional factor for bulk. This modified index is in use to identify patients who require aggressive therapy from the time of diagnosis of their HD (Proctor *et al.*, 1992).

Having identified poor risk patients in our population an aggressive chemotherapeutic regime was formulated for them which included intensification with autotransplant in first remission. A continuous hybrid chemotherapy schedule (PVACE-BOP) was evolved (Proctor *et al.*, 1992) and the details of this are shown in Figure I. The majority of patients in the present study, who underwent ABMT in first or subsequent remission, received this therapy as either first or second line treatment prior to high dose chemotherapy with autotransplant (Table II).

Classical BEAM (BCNU, etoposide, melphalan, cytosine arabinoside) (Gribben *et al.*, 1989) or CBV (cyclophosphamide, carmustine and etoposide) (Armitage *et al.*, 1991; Reece *et al.*, 1991; Jagannath *et al.*, 1989) ablative chemotherapy as preconditioning was considered inappropriate as intensification for patients in 1st CR because of the known toxicity and associated mortality. Our aim in these patients is to attack the minimal residual disease which may not required the same degree of chemotherapeutic intensity developed for treating patients in later stages of disease.

Non-cryopreserved marrow rescue was used as this is associated with a lack of major procedural toxicity and rapid engraftment (Carey *et al.*, 1991; Russell *et al.*, 1989; Köppler *et al.*, 1992). The preconditioning consisted of melphalan and etoposide (VP16), whose short half-life made cryopreservation unnecessary. Patients have not experienced any major toxicity to date.

To conclude, we believe that we have evolved a logical sequence of treating those patients we consider are at high risk of dying from progressive HD in the first 4 years from diagnosis. We therefore:

(1) Identify the poor risk population using the SNLG prognostic index.

PVACE-BOP

Continuous schedule for aggressive Hodgkin's disease
and relapsed progressive Hodgkin's disease

| | |
|---|---|
| Day 1 | Vincristine 2 mg |
| Day 1 | Etoposide IV 100 mg $m^{-2}$ × 1 |
| Day 2,3 | Etoposide Oral 200 mg $m^{-2}$ |
| Day 1–14 | Procarbazine 100 mg $m^{-2}$ |
| Day 1–14 | Chlorambucil 6 mg $m^{-2}$ (max 10 mg) |
| Day 28 | = Day 1 of next course |

| | |
|---|---|
| Day 8 | Doxorubicin 25 mg $m^{-2}$ |
| Day 8 | Vinblastine 6 mg $m^{-2}$ (max 10 mg) |
| Day 14 | Bleomycin 6 mg $m^{-2}$ (max 10 mg) |
| Day 21 | Bleomycin 6 mg $m^{-2}$ (max 10 mg) |
| Day 14–28 | Prednisolone 40 mg daily |

(Proctor *et al.*, 1992)

**Figure 1**

(2) Treat the poor risk group with aggressive chemotherapy from the outset, utilising an eight drug regime which is given continuously for 12 weeks (PVACE-BOP) rather than CLVPP.

(3) Utilise high dose intensification with melphalan/VP16 and ABMT, using pilot information on toxicity and efficacy reported above.

In this way the 40–50% of patients with Stage III or IV Hodgkin's disease who are destined to be cured of their disease with a four drug regimen alone, are not overtreated, and, therefore, are not at increased risk of secondary complications of therapy.

The question of whether patients at risk of early relapse will benefit from high dose chemotherapy with ABMT following intensive primary therapy still remains. Having provided this pilot information on the toxicity and efficacy of ABMT for the SNLG, there is now a prospective randomised trial in progress to try and answer this question (Proctor *et al.*, 1992).

### References

ARMITAGE, J.O., BIERMAN, P.J., VOSE, J.M., ANDERSON, J.R., WEISENBURGER, D.D., KESSINGER, A., REED, E.C., VAUGHAN, W.P., COCCIA, P.F. & PURTILO, D.T. (1991). Autologous bone marrow transplantation for patients with relapsed Hodgkin's disease. *Am. J. Med.*, **91**, 605–611.

BLUME, K.G., FORMAN, S.J. O'DONNELL, M.R., DOROSHOW, J.H., KRANCE, R.A., NADEMANEE, A.P., SNYDER, D.S., SCHMIDT, G.M., FAHEY, J.L., METTER, G.E., HILL, L.R., FINDLEY, D.O. & SNIECINSKI, I.J. (1987). Total body irradiation and high-dose etoposide: a new preparatory regimen for bone marrow transplantation in patients with advanced hematologic malignancies. *Blood*, **69**, 1015–1020.

BONADONNA, G., VALAGUSSA, P. & SANTORO, A. (1986). Alternating non-cross-resistant combination chemotherapy or MOPP in stage IV Hodgkin's disease. *Ann. Int. Med.*, **104**, 739–746.

CAREY, P., PROCTOR, S.J., TAYLOR, P., HAMILTON, P.J. & ON BEHALF OF THE NORTHERN REGIONAL BONE MARROW TRANSPLANT GROUP. (1991). Autologous bone marrow transplantation for high grade lymphoid malignancy using Melphalan/irradiation conditioning without marrow purging or cryopreservation. *Blood*, **77**, 1593–1598.

GRIBBEN, J.G., LINCH, D.C., SINGER, C.R.J., MCMILLAN, A.K., JARRETT, M. & GOLDSTONE, A.H. (1989). Successful treatment of refractory Hodgkin's disease by high-dose combination chemotherapy and autologous bone marrow transplantation. *Blood*, **73**, 340–344.

JAGANNATH, S., DICKE, K.A., ARMITAGE, J.O., CABANILLAS, F.F., HOROWITZ, L.J., VELLEKOOP, L., ZANDER, A.R. & SPITZER, G. (1986). High-dose cyclophosphamide, carmustine and etoposide and autologous bone marrow transplantation for relapsed Hodgkin's disease. *Ann. Intern. Med.*, **104**, 163–168.

JAGANNATH, S., ARMITAGE, J.O., DICKE, K.A., TUCKER, S.L., VELASQUEZ, W.S., SMITH, K., VAUGHAN, W.P., KESSINGER, A., HORWITZ, L.J. HAGEMEISTER, F.B., MCLAUGHLIN, P., CABANILLAS, F. & SPITZER, G. (1989). Prognostic factors for response and survival after high-dose cyclophosphamide, carmustine and etoposide with autologous bone marrow transplantation for relapsed Hodgkin's disease. *J. Clin. Oncol.*, **7**, 179–185.

KLIMO, P. & CONNORS, J.M. (1988). An update on the Vancouver experience in the management of advanced Hodgkin's disease treated with the MOPP/ABV hybrid program. *Semin. Hematol.*, **25**, 34–40.

KÖPPLER, H., PFLÜGER, K.-H., KLAUSMANN, M. & HAVEMANN, K. (1992). High-dose cyclophosphamide, etoposide and BCNU with non-cryopreserved autologous bone marrow transplantation for poor prognosis malignant lymphom. *Leukemia & Lymphoma*, **6**, 219–222.

LENNARD, A., CAREY, P., JACKSON, G. & PROCTOR, S. (1990). An effective oral combination in advanced relapsed Hodgkin's disease: Prednisolone, etoposide, chlorambucil and lomustine (PECC). *Can. Chemo. Pharm.*, **26**, 301–305.

PROCTOR, S.J., TAYLOR, P., THOMPSON, R.B., FINNEY, R., REID, M.M., HAMILTON, P.J., SAUNDERS, P.J., FAIL, B., DICKINSON, A., PAUL, B., QURESHI, M., TINEGATE, H., LENNARD, A., STAINSBY, D., GOFF, D., KAY, L., CARTNER, R., MAMOOD, A., CONDIE, P., COLLINS, A., ABELA, M., RENWICK, L., WALKER, W. & EVANS, R.G.B. (1985). Acute lymphoblastic leukaemia in the Northern Region of England -a study of 75 cases. *Quart. J. Med.*, **57**, 761–774.

PROCTOR, S.J., HAMILTON, P.J., TAYLOR, P., CAREY, P., HARGRAVE, S., EVANS, R.G.B., SUMMERFIELD, G., FINNEY, R., SAUNDERS, P., GOFF, D. & REID, M.M. (1988). A comparative study of combination chemotherapy versus marrow transplant in first remission in adult acute lymphoblastic leukaemia. *Br. J. Haematol.*, **69**, 35–39.

PROCTOR, S.J., TAYLOR, P., DONNAN, P., BOYS, R., LENNARD, A.L., PRESCOTT, R.J. & WITH MEMBERS OF THE SNLG THERAPY WORKING PARTY. (1991). A numerical prognostic index for clinical use in identification of poor risk patients with Hodgkin's disease at diagnosis. *Eur. J. Cancer*, **27**, 624–629.

PROCTOR, S.J., TAYLOR, P., MACKIE, M.J., DONNAN, P., BOYS, R., LENNARD, A., PRESCOTT, R.J. & WITH MEMBERS OF THE SCOTLAND AND NEWCASTLE LYMPHOMA GROUP (SNLG) THERAPY WORKING PARTY. (1992). A numerical prognostic index for clinical use in identification of poor-risk patients with Hodgkin's disease at diagnosis. *Leukemia & Lymphoma*, **7** (Suppl), 17–20.

REECE, D.E., BARNETT, M.J., CONNORS, J.M., FAIREY, R.N., GREER, J.P., HERZIG, G.P., HERZIG, R.H., KLINGEMANN, H.-G., O'REILLY, S.E., SHEPHERD, J.D., SPINELLI, J.J., VOSS, N.J., WOLFF, S.N. & PHILLIPS, G.L. (1991). Intensive chemotherapy with cyclophosphamide, carmustine, and etoposide followed by autologous bone marrow transplantation for relapsed Hodgkin's disease. *J. Clin. Oncol.*, **9**, 1871–1879.

RUSSELL, J.A., SELBY, P.J., RUETHER, B.A., MBIDDE, E.K., ASHLEY, S., ZULIAN, G., BERRY, J., HOUWEN, B., JONES, A.R., POON, M.-C., BLAHEY, W.B., BRADA, M., NANDI, A., GEGGIE, P.H.S., GORE, M.E. & MCELWAIN, T.J. (1989). Treatment of advanced Hodgkin's disease with high dose melphalan and autologous bone marrow transplantation. *B.M. Transplant*, **4**, 425–429.

STEWART, A.K., BRANDWEIN, J.M., SUTCLIFFE, S.B., SCOTT, J.G. & KEATING, A. (1991). Mini-beam as salvage chemotherapy for refractory Hodgkin's disease and non-Hodgkin's lymphoma. *Leukemia & Lymphoma*, **5**, 111–115.

WOLFF, S.N., FER, M.F., & MCKAY, C.M. (1983). High dose VP-16-213 and autologous bone marrow transplantation for refractory malignancies: a phase I study. *J. Clin. Oncol.*, **1**, 701.

WORLD HEALTH ORGANIZATION (1979). WHO Handbook for Reporting Results of Cancer Treatment, World Health Organisation: Geneve.

ZULIAN, G.B., SELBY, P., MILAN, S., NANDI, A., GORE, M., FORGESON, G., PERREN, T.J. & MCELWAIN, T.J. (1989). High dose melphalan, BCNU and etoposide with autologous bone marrow transplantation for Hodgkin's disease. *Br. J. Cancer*, **59**, 631–635.